# Action simulation in hallucination-prone adolescents

**DOI:** 10.3389/fnhum.2013.00329

**Published:** 2013-07-04

**Authors:** Tarik Dahoun, Stephan Eliez, Fei Chen, Deborah Badoud, Maude Schneider, Frank Larøi, Martin Debbane

**Affiliations:** ^1^Office Médico-Pédagogique Research Unit, Department of Psychiatry, University of Geneva School of MedicineGeneva, Switzerland; ^2^Department of Genetic Medicine and Development, University of Geneva School of MedicineGeneva, Switzerland; ^3^Adolescence Clinical Psychology Research Unit, Faculty of Psychology and Educational Sciences, University of GenevaGeneva, Switzerland; ^4^Department of Psychology, University of LiègeLiège, Belgium

**Keywords:** auditory hallucinations, 22q11.2, action simulation, perspective-taking

## Abstract

Theoretical and empirical accounts suggest that impairments in self-other discrimination processes are likely to promote the expression of hallucinations. Studies using a variety of paradigms involving self-performed actions argue in favor of perspective taking confusion in hallucination-prone subjects. However, our understanding of such processes during adolescence is still at an early stage. The present study thus aims (1) to delineate the neural correlates sustaining mental simulation of actions involving self-performed actions (first-person perspective; 1PP) and other-performed actions (third-person perspective; 3PP) during adolescence (2) to identify atypical activation patterns during 1PP/3PP mental simulation of actions in hallucination-prone adolescents (3) to examine whether differential risk for schizophrenia (clinical vs. genetic) is also associated with differential impairments in the 1PP/3PP mental simulation of actions during adolescence. Twenty-two typically developing controls (Control group; 6 females), 12 hallucination-prone adolescents [auditory hallucination (AH) group; 7 females] and 13 adolescents with 22q11.2 Deletion Syndrome (22q11.2DS group; 4 females) were included in the study. During the fMRI task, subjects were presented with a cue (self-other priming cues) indicating to perform the task using either a first person perspective (“you”-1PP) or a third person perspective (“best friend”-3PP) and then they were asked to mentally simulate actions based on the type of cue. Hallucination-proneness was assessed using a self-report questionnaire [Cardiff Anomalous Perception Scale (CAPS)]. Our results indicated that atypical patterns of cerebral activation, particularly in the key areas of self-other distinction, were found in both groups at risk for auditory hallucinations (AHs and 22q11.2DS). More precisely, adolescents in the AH group presented decreased activations in the right middle occipital gyrus BA19, left cingulate gyrus BA31, and right precuneus BA31 for the 3PP > 1PP contrast. Adolescents in the 22q11.2DS group presented decreased activations in the right superior occipital gyrus BA19, left caudate tail and left precuneus BA7 for the 3PP > 1PP contrast. In comparison to the Control group, only the 22q11.2DS adolescents showed a decreased activation for other-related cues (prime other > prime self contrast) in areas of visual imagery, episodic memory and social cognition. This study characterizes the neural correlates of mental imagery for actions during adolescence, and suggests that a differential risk for hallucination-proneness (clinical vs. genetic) is associated to similar patterns of atypical activations in key areas sustaining self-other discrimination processes. These observations may provide relevant information for future research and prevention strategies with regards to hallucination-proneness during adolescence.

## Introduction

Auditory hallucinations (AHs) have been conceptualized as a neurodevelopmental phenomenon (Bentall et al., 2007) with a prevalence varying from 6 to 33% in adolescence (see review Larøi et al., 2006). A number of cognitive processes are thought to sustain the expression of AH, such as attention shift/enhancement, executive and inhibitory deficits, and source monitoring (SM) (Hugdahl, 2009; Jones, 2010; Badcock and Hugdahl, 2012; Waters et al., 2012). The developmental course of these cognitive processes during childhood and adolescence suggests that investigating hallucination-proneness during these key developmental windows may help better understand the onset of early AH.

SM is a processes associated with the development of AH. Impairments in SM are thought to lead to the misattribution of self-generated mental contents such as thoughts, memories or action to external sources in hallucination-prone adults (Bentall and Slade, 1985; Rankin and O'Carroll, 1995; Larøi et al., 2004, 2005), in adults with schizophrenia (Bentall et al., 1991; Rankin and O'Carroll, 1995; Blakemore et al., 2000; Brebion et al., 2000; Brunelin et al., 2006) and in adolescents at high genetic risk for psychosis (22q11.2 Deletion Syndrome; Debbane et al., 2010).

Most of these studies used a verbal SM paradigm. However Larøi and collaborators used a SM task for actions and found evidence for misattribution of imagined actions in hallucinations-prone adults (Larøi et al., 2005). In this study, subjects were asked to (1) imagine themselves or (2) the experimenter performing an action (3) repeat the action statement without imaging the action or (4) simply observe the experimenter carrying out the action. The results revealed that hallucination-prone subjects more often remembered self-performed imagined actions as being imagined actions performed by the experimenter.

A subsequent study employed a similar action-monitoring paradigm with adolescents affected by a 22q11.2 deletion syndrome (22q11.2DS) (Debbane et al., 2008). 22q11.2DS is a neurogenetic disorder with an ultra-high risk for developing schizophrenia (Murphy et al., 1999; Karayiorgou et al., 2010). Transient psychotic experiences are characteristic of more than half of the adolescents with this syndrome (Baker and Skuse, 2005). Furthermore, AHs are the most commonly reported symptoms in the sample of 22q11.2DS children and adolescents investigated by our group (Debbane et al., 2006). The assessment of 22q11.2DS adolescents with a SM task adapted from Larøi et al. (2005), showed that adolescents with 22q11.2DS committed more source confusions by recalling imagined-experimenter actions as actions they had mentally repeated (and vice versa), suggesting potential impairments in third person perspective (3PP) taking.

These two studies (Larøi et al., 2005; Debbane et al., 2008) highlight the impairments in *offline* SM for actions in two populations with hallucination-proneness. Their results might come from disturbances in how information is encoded between first-person perspective (1PP) and 3PP. Theoretical explanations suggest that encoding processes during *online* representation of actions may promote subsequent confusion between self and other by two complementary aspects (1) increased salience of internal representations leading to exaggerated self-focused orientation (Ingram, 1990; Ensum and Morrison, 2003; Kapur, 2003; Perona-Garcelan et al., 2011) (2) impairments in the sense of agency, i.e., the ability to experience oneself as the agent of one's own actions (Gallagher, 2000), as evoked by several authors (Schneider, 1959; Seal et al., 2004; Jones and Fernyhough, 2007; Asai and Tanno, 2012). Among the multiple neurocognitive models of the sense of agency (David et al., 2008; Sperduti et al., 2011; Gallagher, 2012), Jeannerod and colleagues propose to differentiate between actions overtly executed and those that remain covert, i.e., internally represented (Jeannerod, 1994; Georgieff and Jeannerod, 1998). Self-other attribution of covert actions might be sustained by the activity of brain areas specifically devoted to self-other representations (Georgieff and Jeannerod, 1998; Jeannerod and Pacherie, 2004; Jeannerod, 2006).

In order to identify the specific regions involved in the discrimination of self-other action simulation, Ruby and Decety (2001) employed positron emission tomography (PET) to compare the neural correlates of action simulation in a 1PP and a 3PP. Their results showed that both 1PP and 3PP involve overlapping areas of neural processing, in accordance with the shared neural representations theory (Georgieff and Jeannerod, 1998; Grezes and Decety, 2001; Decety and Chaminade, 2003; Decety and Sommerville, 2003). However, specific regions were identified in the right inferior parietal, precuneus, posterior cingulate and frontopolar cortices for 3PP, and in the left inferior parietal and somatosensory cortices for 1PP. The authors concluded that the right inferior parietal, precuneus and somatosensory cortices are key areas involved in self/others action discrimination. The inferior parietal lobule is thought to be involved in body image, self-recognition and integration of information coming from sensory modalities and proprioceptive signals (Jeannerod and Pacherie, 2004; Torrey, 2007). Interestingly, increased activation in the inferior parietal lobule has been observed during conflict between a self-produced action and its consequences (Farrer et al., 2003). The anterior region of the precuneus is related to self-centered imagery and the posterior part to successful episodic memory retrieval (Cavanna and Trimble, 2006). According to the authors, the somatosensory cortex could play a role in self-representation (Ruby and Decety, 2001, 2003, 2004).

Another study focused more specifically on the visuo-spatial aspects of perspective taking during action imagery (Jeannerod and Anquetil, 2008). The authors compared brain activity with PET while subjects imagined the same action (reaching and grasping a cylinder) from a 1PP and 3PP. This paradigm revealed increased activation in the parieto-occipital junction (BA19) specifically for the 3PP. The authors conclude that the right BA 19 is a key area for self-other differentiation by evaluating the difference in spatial localization between oneself and an other's perspective.

In summary key areas of self/other distinction for covert actions are thought to essentially engage the parietal cortex region for multi-modal integration and the parietal-temporal-occipital region, which underpins the shift to another location in space during perspective taking (Ruby and Decety, 2001, 2003, 2004; Vogeley and Fink, 2003; Jeannerod, 2004; David et al., 2007).

In order to investigate the neural correlates underlying both self- and other-focused orientation and self-other perspective taking during action imagery, we used a functional magnetic resonance imagery paradigm adapted from Larøi et al. (2005). During this task subjects were first primed with a self-other priming cues (namely “you” or “best friend”) and secondly were asked to mentally simulate actions with either from a first-person (1PP) or a third-person (best friend) perspective (3PP) in accordance with the priming cue. Typically developing adolescents and adolescents clinically prone to hallucinate (AH group) as well as with a 22q11.2 deletion syndrome (22q11.2DS group) underwent this task.

This study has three aims: (1) delineate the neural correlates of action simulation in specific 1PP and 3PP during adolescence (2) identify potential impairments at a neurofunctional level in hallucination-prone adolescents (3) examine whether a differential risk for schizophrenia (clinical vs. genetic) is also associated with differential impairments in the mental simulation of action during adolescence.

We hypothesized that: (1) typically developing adolescents would activate specific regions devoted to 1PP and 3PP already observed in adult subjects (Ruby and Decety, 2001; Jeannerod and Anquetil, 2008) (2) the AH group and the 22q11.2DS group would present atypical patterns of brain activation in regions sustaining self-other action simulation, along with confusion in self-other remembered actions. (3) Subjects with 22q11.2DS would present atypical patterns of activation in the parietal cortices due to functional and structural impairments (Simon et al., 2005b; Dufour et al., 2008; Bearden et al., 2009; Schaer et al., 2010; Debbane et al., 2012) whereas the AH group would exhibit atypical activations in the prefrontal cortex, as suggested by previous SM studies (Vinogradov et al., 2008; Lagioia et al., 2011; Wang et al., 2011).

## Materials and methods

### Participants

Eighty adolescents aged from 12 to 20 years participated in the study. Exclusion criteria included age, the presence of any neurological problem or a diagnosis of schizophrenia or schizoaffective disorder according to DSM-IV-TR criteria. Thirty-two subjects were excluded for head movement exceeding 4.7 mm in any of the 6 directions during the scan sessions (Control group: *N* = 9, AH group: *N* = 6, 22q11.2DS group: *N* = 17). In the Control group, we excluded subjects with maladaptive functioning above the clinical cut-off of the Internalizing and Externalizing scales (*t*-score >64) in the Youth Self-Report and Adult Behavior Checklist (Achenbach, 1991, 1997) (*N* = 1). After excluding these 33 subjects, the 47 remaining youths were distributed in the following three groups: typically developing adolescents (Control group: *N* = 22), adolescents with transient AHs (AH group: *N* = 12) and adolescents with a 22q11.2 Deletion Syndrome (22q11.2DS group: *N* = 13).

Out of the 22 subjects in the Control group (mean age: 16.00, *SD* = 2.04, 16 males), 6 were recruited within the siblings of 22q11.2DS participants and 16 from the Geneva state school system.

In the AH group, 12 subjects with subclinical AHs (mean age: 15.97, *SD* = 2.12, 5 males) were recruited through patient associations, by word of mouth or through the Child and Adolescents Outpatient Public Service (Office Médico-Pédagogique). Subjects were selected on the basis of a positive answer (yes or no) on the Cardiff Anomalous Perceptions Scale (CAPS) items describing AH items [i.e., items 3, 7, 11, 13, 28, or 32; (Bell et al., 2006; Debbané et al., 2011); see Table 1]. If they answered positively to an item, they were asked to rate their distress, the intrusiveness and the frequency of the experience by circling a number between 1 (not at all) and 5 (very).

**Table 1:** **CAPS selected items for auditory hallucinations (Bell et al., 2006; Debbané et al., 2011)**.

Item 3: “Do you ever hear your own thoughts repeated or echoed?”				
Item 7: “Do you ever hear your own thoughts spoken aloud in your head, so that someone near might be able to hear them?”				
Item 11: “Do you ever hear voices commenting on what you are thinking or doing?”				
Item 13: “Do you ever hear voices saying words or sentences when there is no one around that might account for it?”				
Item 28: “Have you ever heard 2 or more unexplained voices talking with each other?”				
Item 32: “Do you ever hear sounds or music that people near you don't hear?”				
	**CAPS mean sum selected items**	**CAPS mean selected items distress**	**CAPS mean selected items frequency**	**CAPS mean selected items intrusiveness**
Control group	0	N/A	N/A	N/A
AH group	2.25 (1.91)	2.66 (1.25)	1.77 (0.66)	2.78 1.10
22q11.2DS group	0.54 (0.47)	2.71 (1.89)	2.79 (1.81)	2.38 (1.60)

In the 22q11.2DS group, all adolescents (mean age: 16.14, *SD* = 2.55, 9 males) were recruited through parent associations in France, Belgium and Switzerland. The 22q11.2 deletion was confirmed using DNA polymorphism analysis based on short sequence repeats or by fluorescence in situ hybridization performed on metaphase spreads spanning the deleted region.

Written informed consent was accepted by all parents and/or subjects under protocols approved by the Institutional Review Board of the Geneva University School of Medicine. The three groups (Control, AH and 22q11.2DS) did not significantly differ according to age and gender (*p* > 0.05). At the time of testing, no participants were receiving psychotropic medication (data for this was missing for one subjects in the 22q11.2DS group). All participants underwent the Block Design subtest (Kohs, 1920) in order to assess intellectual scores.

### Design and procedure

**Figure 1:**
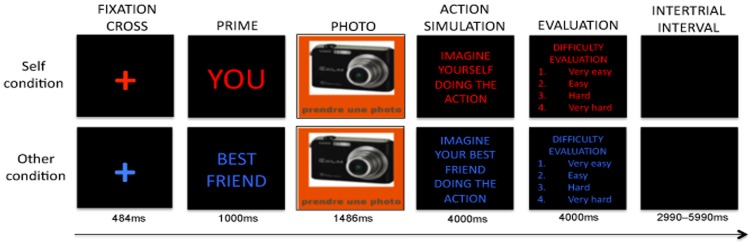
**Action simulation task adapted from Larøi et al. (2005)**.

Before the scan session, the experimenter described the task to the participants (see Figure 1). The paradigm was adapted from Larøi et al. (2005) and included 60 actions to be mentally simulated (imagined) either from a 1PP or 3PP. Simple, universal and gender-neutral actions were chosen. All actions implied a movement and an object (for example *take a picture, open a bottle, open a window, play the violin, brush your hair*). 30 actions were tested with a 1PP and 30 with a 3PP, in the same randomized order for each participant.

At the start of each session, the task's instructions appeared on the screen as a reminder. Then, a cross appeared on the screen for 665 ms. Immediately after the self-other priming cue appeared for 1 s stating either “You” written in red or “Best friend” in blue. Then, the photo of an object accompanied by a written instruction specifying the action to be imagined (i.e., play the violin, open a bottle, knock on a door) appeared for 1487 ms. Then the participants were reminded to either “Imagine yourself doing the action” or “Imagine your best friend doing the action” in accordance with the self-other priming cue. This instruction remained on the screen for a total of 4 s. An instruction in the center of the screen asked participants were asked to evaluate the difficulty of imagining the previous action, by pressing 1 (very easy) to 4 (very hard) on a set of buttons on a console. This last step was used to make sure that the subjects performed the task. When a button was pressed, a blank screen appeared followed by the inter-trial interval (ITI) period, which lasted from 2990 to 5990 ms.

### fMRI data acquisition

A 3T Siemens TIM Trio system was used to acquire anatomical and functional images [*TR* (inter-trial between scan acquisition) = 2400 ms, Echo time (*TE*) = 30 ms, Slice thickness = 3.20 mm, Flip angle = 85°, FOV 235 mm]. The functional scan session consisted of 380 volumes that comprised 38 slices oriented parallel to the AC-PC lines and collected in a descending sequence. High-resolution three-dimensional anatomical images were also obtained [*TR* (inter-trial between scan acquisition) = 2400 ms, *TE* = 30 ms, Slice thickness = 1.1 mm, Flip angle = 8°, 192 coronal slices, FOV 220 mm].

### fMRI data analysis

We used Statistical Parametric Mapping (SPM) 8, (Welcome Department of Neuroscience, London, UK) to analyse the data. First of all images had to be spatially transformed during the pre-processing step in order to reduce movement effects or shape differences among a series of scans. We realigned every image with respect to the first one. Then, slice timing correction was performed using the middle slice as a reference. We co-registered structural images of each participant to the mean of the realigned functional images. Gray matter separation was established by segmentation of the anatomical image. Thereafter, the normalization produced images that were warped to fit to a standard Template brain. We normalized the realigned and slice-timed images into the Montreal Neurological Institute (MNI) template using 3 × 3 × 3 mm isotropic voxels. The images were spatially smoothed with an isotropic Gaussian smoothing Kernel of 6 mm full width half maximum (FWHM) to conform to inter-individual brain size variability.

After pre-processing, the brain responses of each subject were estimated at every voxel using a general linear mode. We defined two main conditions namely “self” and “other.” The “self” condition corresponds to the trials starting with the word “you” in the priming period and when imagining an action performed by oneself (action stimulation period). The “other” condition refers to the trials starting with the word “best friend” in the priming period and when imagining an action performed by the best friend (action stimulation period). The return to baseline periods were set in the ITIs during which subjects saw a blank screen for 2990 ms to 5990 ms between each trial. In order to compare the specific areas devoted to the two different periods of the task (prime period and action simulation period) voxel value maps of t statistics were obtained for 4 contrasts: (1) prime self > prime other (2) prime other > prime self (3) 1PP > 3PP (4) 3PP > 1PP. These contrasts were performed for the following reasons. First, the prime self > prime other contrast will shield information on the neural correlates related to attention oriented to the self. In relation to our knowledge about overlapping activated brain regions for 1PP and 3PP-taking, some authors have suggested that 3PP requires “additional” areas in contrast to 1PP and vice-versa (Jeannerod, 2004, 2006; Jeannerod and Pacherie, 2004; Jeannerod and Anquetil, 2008). As such, it may be that hallucination-prone subjects fail to properly engage these areas, thereby increasing possible confusions between self and other. The two other contrasts (1PP > 3PP, 3PP > 1PP) follow the same logic, but when considering actual action imagery. T-maps were produced to identify atypical activation of the neural correlates sustaining self-other orientation and perspective taking for actions.

We first performed a one-sample *t*-test to characterize typical activations in control adolescents, and then proceeded to group comparison analyses. Using a two-sample *t*-test (comparison between groups), we compared Control and AH groups, Control and 22q11.2DS groups, and finally 22q11.2DS and AH groups. S{T} maps were obtained with a threshold of *p* < 0.05 and an extend threshold k of 20 voxels. Cluster level peak functional activity at *p* < 0.05 (Family-Wise corrected) was then localized on a mean structural scan with approximate Brodmann areas estimated from the Talairach and Tournoux (1988) atlas after having converted coordinates from MNI to Talairach templates (http://www.bioimagesuite.org/Mni2Tal/index.html). Age was entered as a covariate in each analysis without any significant effects on the results obtained.

For *post-hoc* examination of potential associations between hallucination-proneness scores and activations resulting from group comparisons, we planned to extract local brain activity of regions of interest (ROIs) using SPM8 toolbox Marsbar (http://marsbar.sourceforge.net/). The ROIs were delimited around the peak of significant activations in prime self > prime other, prime other > prime self, 1PP > 3PP, 3PP > 1PP contrasts for the group comparisons. A 5 mm radius sphere was defined around the center of mass for each subject to extract Beta Values. We performed Pearson correlations between Beta Values obtained for different ROIs and CAPS components for each subjects (AHs distress, intrusiveness, frequency, total scores, as well as subscale scores) (Bell et al., 2006; Debbané et al., 2011).

## Results

### Behavioral results

Differences regarding evaluation results between the three groups and the two different conditions were analysed using a repeated-measures ANOVA 3(groups) × 2(conditions) with *post-hoc* Tukey analyses.

With regard to the evaluation of difficulty ratings (see Table 2), our 3 × 2 ANOVA yielded a non-significant effect of diagnosis [*F*_(2, 44)_ = 1.875, *p* = 0.165], a significant effect of condition [*F*_(1, 44)_ = 13.315, *p* = 0.001^***^] (mean evaluation for self condition = 1.6820, *SD* = 0.40360), (mean evaluation for other condition = 1.8504, *SD* = 0.47230) and a non-significant interaction between diagnosis and conditions [*F*_(2, 44)_ = 0.752, *p* = 0.477].

**Table 2:** **Evaluation, response time, and Block DESD in each group**.

	**Control group (*N* = 22)**	**AH group (*N* = 12)**	**22q11.2DS group (*N* = 13)**
Evaluation other	1.72 (0.38)	2.06 (0.45)	1.87 (0.57)
Evaluation self	1.59 (0.33)	1.79 (0.34)	1.74 (0.53)
Evaluation total	1.67 (0.32)	1.92 (0.37)	1.79 (0.53)
Answer time other	1142.24 (446.29)	1082.13 (294.93)	1118.20 (351.85)
Answer time self	1135.46 (425.56)	992.64 (266.31)	1076.48 (306.47)
Answer time total	1138.93 (425.45)	1037.40 (261.78)	1097.20 (303.49)
Block DESD	11.5 (3.25)	11.75 (2.53)	4.86 (2.73)

With regard to response time (see Table 2), results yielded a non-significant effect of diagnosis [*F*_(2, 44)_ = 0.319, *p* = 0.729], a non-significant effect of condition [*F*_(1, 44)_ = 2.054, *p* = 0.159] and a non-significant interaction between diagnosis and condition [*F*_(2, 44)_ = 0.593, *p* = 0.557].

### Neuroimaging results

#### Control group

***Prime period.*** The prime other > prime self contrast was associated with activations in a first cluster (2623 voxels, *p* = 0.001), including significant activations in the right cuneus BA18, right posterior cingulate BA30 and left cuneus BA17 (see Table 3). A second cluster (2272 voxels, *p* = 0.004) included significant activations in the right superior frontal gyrus BA6, left middle frontal gyrus BA46 and right superior frontal gyrus BA6 (see Table 3). No significant results were obtained in the prime self > prime other contrast.

**Table 3:** **Regions of peak activations in the Control group**.

**Contrast**	**Cluster level - *p* -FEW-corr**	**Cluster level - Ke - voxels**	**Side**	**Brain regions activation**	**Brodmann area**	***T*-value**	***X, Y, Z* (MNI)**
Prime other > Prime self	0.001	2623	Right	Occipital lobe, cuneus	BA18	5.10	3, −76, 19
	0.001		Right	Limbic lobe, posterior cingulate	BA30	5.06	9, −67, 10
	0.001		Left	Occipital lobe, cuneus	BA17	4.72	−21, −82, 13
	0.004	2272	Right	Frontal lobe, superior frontal gyrus	BA6	5.04	6, 32, 64
	0.004		Left	Frontal lobe, middle frontal gyrus	BA46	4.07	−51, 29, 19
	0.004		Right	Frontal lobe, superior frontal gyrus	BA6	3.90	21, 26, 64
3PP > 1PP	0.000	4098	Right	Limbic lobe, cingulate gyrus	BA23	4.90	3, −31, 28
	0.000		Right	Occipital lobe, cuneus	BA18	4.55	6, −73, 16
	0.000		Left	Occipital lobe, middle occipital gyrus	BA18	4.52	−21, −85, 16
	0.025	1692	Left	Frontal lobe, precentral gyrus	BA6	3.69	−39, 2, 40
	0.025		Left	Frontal lobe, superior frontal gyrus	BA6	3.68	−3, 17, 67
	0.025		Left	Frontal lobe, superior frontal gyrus	BA9	3.57	−18, 41, 43

***Action simulation period.*** The 3PP > 1PP contrast was associated with activations in a first cluster (4098 voxels, *p* = 0.000) including activations in the right cingulate gyrus BA23, right cuneus BA18 and left middle occipital gyrus BA18. A second cluster (1692 voxels, *p* = 0.025) included significant activations in the left precentral gyrus BA6, left superior frontal gyrus BA6 and left superior frontal gyrus BA9. No significant results were obtained in the 1PP > 3PP contrast.

### Neuroimaging results: group comparisons

#### Comparisons between control and AH groups

***Action simulation period.*** We observed significant results for the Control > AH comparison in the action simulation period (see Table 4). Specifically, the 3PP > 1PP contrast was associated with activations in a single cluster (5569 voxels, *p* = 0.000) including significant activations in the left middle occipital gyrus BA19, left cingulate gyrus BA31 and in the right precuneus BA31.

**Table 4:** **Regions of peak activations for group comparisons Control > AH**.

**Contrast**	**Cluster level - *P* FEW-corr**	**Cluster level - Ke - voxels**	**Side**	**Brain regions activation**	**Brodmann area**	***T*-value**	***X, Y, Z* (MNI)**
3PP > 1PP	0.000	5569	Right	Occipital lobe, middle occipital gyrus	19	4.42	33, −76, 19
	0.000		Left	Limbic lobe, cingulate gyrus	31	4.17	0, −37, 31
	0.000		Right	Occipital lobe, precuneus	31	4.09	24, −79, 31

#### Comparisons between control and 22q11.2DS groups

***Prime period.*** We observed significant results for the Control > 22q11.2DS comparison (see Table 5). The prime other > prime self contrast was associated with activations in a single cluster (2716 voxel, *p* = 0.001) with significant activations in the left cuneus BA18, left precuneus BA31, right middle temporal gyrus BA39.

**Table 5:** **Regions of peak activations for group comparisons Control > 22q11.2DS**.

**Contrast**	**Cluster level - *P* FEW-corr**	**Cluster level - Ke - voxels**	**Side**	**Brain regions activation**	**Brodmann area**	***T*-value**	***X, Y, Z* (MNI)**
Prime other > Prime self	0.001	2716	Left	Occipital lobe, cuneus	BA18	4.52	−6, −82, 19
	0.001		Left	Parietal lobe, precuneus	BA31	4.13	−18, −73, 25
	0.001		Right	Temporal lobe, middle temporal gyrus	BA39	3.98	30, −67, 22
3PP > 1PP	0.000	7020	Right	Occipital lobe, superior occipital gyrus	BA19	5.37	36, −76, 25
	0.000		Left	Sub-lobar, caudate, caudate tail		4.93	−18, −25, 19
	0.000		Left	Parietal lobe, precuneus	BA7	4.92	−21, −73, 31

***Action simulation period.*** The 3PP > 1PP contrast was associated with activations in a single cluster (7020 voxels, *p* = 0.000) with significant activations in the right superior occipital gyrus BA19, left caudate tail and left precuneus BA7 (see Table 5).

#### Comparisons between 22q11.2DS and AH

***Prime period.*** We observed significant results for the AH > 22q11.2DS comparison (see Table 6). The prime self > prime other contrast was associated with activations in a single cluster (1468 voxels, *p* = 0.041) with significant activations in the left caudate body, right anterior cingulate gyrus BA32 and right superior frontal gyrus BA 10. The prime other > prime self contrast was associated with activations in a single cluster (1696 voxels, *p* = 0.020) with significant activations in the right postcentral gyrus BA3, left superior frontal gyrus BA10 and right superior frontal gyrus BA8. No significant clusters were detected in the action simulation period.

**Table 6:** **Regions of peak activations for group comparisons AH > 22q11.2DS**.

**Contrast**	**Cluster level - *P* FEW-corr**	**Cluster level - Ke - voxels**	**Side**	**Brain regions activation**	**Brodmann area**	***T*-value**	***X, Y, Z* (MNI)**
Prime self > Prime other	0.041	1468	Left	Sub-lobar, caudate, caudate body		3.79	−12, 26, 16
	0.041		Right	Limbic lobe, anterior cingulate	BA32	3.67	15, 32, −8
	0.041		Right	Frontal lobe, superior frontal gyrus	BA10	3.24	24, 59, 10
Prime other > Prime self	0.020	1696	Right	Parietal lobe, postcentral gyrus	BA3	3.22	66, −19, 37
	0.020		Left	Frontal lobe, superior frontal gyrus	BA10	2.88	−12, 71, 16
	0.020		Right	Frontal lobe, superior frontal gyrus	BA8	2.84	6, 38, 52

#### ROIs analyses

No significant results were obtained for Pearson correlations between *T*-values activations in Control > AH, Control > 22q11.2DS and AH > 22q11.2DS group comparisons and CAPS subscales scores for each subjects.

## Discussion

This study is the first to compare neural correlates in self-other priming cues and action simulation using a 1PP or 3PP in typically developing adolescents (Control group), adolescents with transient AHs (AH group) and adolescents at genetic risk for schizophrenia (22q11.2DS group). The three objectives of this study were (1) to delineate the neural correlates sustaining mental simulation of actions involving 1PP and 3PP during adolescence; (2) to identify potential atypical neural activations during self-other priming and/or action simulation in hallucination-prone adolescents; (3) to examine whether differential risk for hallucination-proneness (clinical vs. genetic) is also associated with differential impairments in self-related cues and in action simulation. Our findings showed that (1) the Control group activated the key areas involved in *other* related cues when primed for their best friend compared to themselves, and in action simulation performed by others; (2) in the 3PP condition both hallucination-prone groups exhibited decreased activation in the parieto-occipital region, which has been related to self-other distinction of imagined actions (Jeannerod and Anquetil, 2008); (3) the priming period for both self and other related cues showed decreased activations in subjects with 22q11.2DS compared to those at clinical risk.

Control group activation patterns during prime and action simulation periods will first be discussed. Then, the unique activations in the AH group and the 22q11.2DS group will be brought into consideration, followed by a discussion concerning the differences between the two hallucination-prone groups.

### Control group

Typically developing adolescents showed significant increased activations for the “other” condition compared to the “self” condition in both the prime and the action simulation periods.

**Figure 2:**
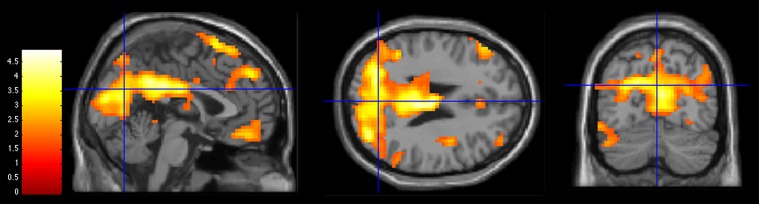
**Activations during 3PP > 1PP contrast in Control group at a statistical threshold of *p* < 0.05**. Slice views at MNI coordinates (*x* = 0, *y* = −70, *z* = 25). The bar on the left shows the range of *T*-values.

For the 3PP, we found increased activations in the PCC and the parieto-occipital regions (see Figure 2). These regions may underlie the influence of visuo-spatial components and episodic memory when adolescents imagine actions performed by their best friend. PCC is involved in the processing of familiar stimuli (Qin and Northoff, 2011; Qin et al., 2012), and it has been shown that it plays an important role in memory tasks such as remembering familiar people (Maddock et al., 2001), remembering familiar objects and places (Sugiura et al., 2005) and autobiographical memory (Summerfield et al., 2009; Van Der Meer et al., 2010). The mental simulation of actions may involve the retrieval of memorized visual representations (Farah, 1984; Annett, 1995) of the imaginary action.

Areas in the parieto-occipital region lobe are activated when processing visuo-spatial information in the context of action representation (Kilintari et al., 2011), object-distance representation (Berryhill and Olson, 2009), including position and prediction of moving objects (Maus et al., 2010), coherent moving visual motion (McKeefry et al., 1997; Braddick et al., 2001), and motor imagery of hand action (Willems et al., 2009). The 3PP contrast might engage supplementary areas devoted to motion and visuo-spatial information, as an other's perspective implies a shift in visual-spatial perspective (Vogeley et al., 2004).

Activations in the frontal lobe could reflect the self-relevance evaluation of the prime period and the motor cognition aspect of action simulation. For the prime other > prime self contrast, we found significant increased activations in the right superior frontal part of the medial prefrontal cortex and the left dorsolateral prefrontal cortex DLPFC (Mayka et al., 2006; Northoff et al., 2006; Murray et al., 2012). These results are consistent with recent findings on self-other related processes in healthy adults. According to a recent meta-analysis, the dorsomedial prefrontal cortex DMPFC, DLPFC, and PCC act together in the evaluation and decision-making processes of self versus other relevant information (Van Der Meer et al., 2010).

For the action simulation period, we obtained significant results in the ventral part of the dorsal premotor cortex (Grezes and Decety, 2001; Mayka et al., 2006), the left pre-supplementary motor area (pre-SMA) (Mayka et al., 2006) and the DLPFC. These regions could be recruited by the task's motor aspects. It has been shown that the ventral part of the dorsal premotor cortex plays a role in motor preparation (Hoshi and Tanji, 2007), the pre-SMA in maintaining an action representation (Stadler et al., 2011) and the DLPFC in the cognitive control of motor behavior (Passingham, 1993; Hoshi, 2006; Cieslik et al., 2012).

In summary, our results showed that the mental simulation of actions performed by others engage increased activations in the posterior midline structure including PCC and the parieto-occipital region. Our results may reflect the visuo-spatial and episodic memory components of self-other discrimination for imagined actions.

### Similarities and differences for the AH group and the 22q11.2DS group compared to control group

Both hallucination-prone groups showed significant decreased activations for the 3PP > 1PP contrast compared to the control group. The AH group and the 22q11.2DS group presented an atypical pattern of activations in the parieto-occipital region with significant decreased activations in the occipital gyrus BA19 and the precuneus.

As reviewed above, it has been shown that the right superior occipital BA19 is specifically devoted to 3PP in an action imagery task focusing on a visuo-spatial perspective switch (Jeannerod and Anquetil, 2008). According to the authors, the mental simulation of actions performed by others first occurs through a shift in space in order to mentally represent the other's place, and is then followed by the action simulation per se. In this framework, BA 19 would be a key area for self-other distinction by evaluating the difference in spatial localization between oneself and someone else. This interpretation is supported by data demonstrating the role of BA19 in the manipulation of spatial relationships between objects (Haxby et al., 1991; Kosslyn et al., 1998) and further confirmed by a meta-analysis (Zacks, 2008). Clinical studies have also shown that posterior parietal lesions provoke visuo-spatial dysfunction (Mendez, 2001; Harvey and Rossit, 2012) or disturbances in the capacity to represent relative location of objects with respect to the subject (Aguirre and D'Esposito, 1999). Recent evidence shows that the parieto-occipital junction responds to both gaze- and body-centered representation when reaching a target visually presented (Bernier and Grafton, 2010). This could be an argument in favor of a gaze and body reference computed by parieto-occipital junction during shift in 3PP.

Concerning the 22q11.2DS, Bearden et al. (2009) interestingly detected an decreased cortical thickness in the right parieto-occipital cortex, while to our knowledge, no clear structural alterations have been identified in this region in hallucination-prone subjects (Allen et al., 2008). Moreover, it has been shown that children with 22q11.2DS tend to present significant decreased activation in the parietal and occipital lobe during a visuo-spatial working memory task (Azuma et al., 2009). From a clinical point of view, visuo-spatial impairments have been extensively reported in the syndrome (Wang et al., 2000, 2011; Simon et al., 2005a; Jacobson et al., 2010). Together, these findings argue in favor of an atypical neuro-development of the parieto-occipital region in 22q11.2DS, which could lead to deficits in visuo-spatial perspective shifting in actions with objects.

The second region showing decreased activation during 3PP > 1PP contrast for both hallucination-prone groups was located in the right precuneus BA 31 (AH group) and the left precuneus BA7 (22q11.2DS group). In the PET study previously mentioned, Ruby and Decety (2001) found stronger activation bilaterally in the precuneus for 3PP > 1PP, and thus considered the region as specifically involved in distinguishing self and other action imagery. According to their view, the precuneus would play a role in the self's representation with an overactivation during 3PP. The precuneus responds to a wide range of cognitive processes including internal self-representation, episodic memory retrieval, visuo-spatial imagery, 1PP and agency processes (Cavanna and Trimble, 2006). The anatomical and connectivity data reviewed by them converges toward a functional subdivision between the anterior (y closer to −60 mm) and posterior (y closer to −70 mm) precuneus (Cavanna and Trimble, 2006). Our results for both the AH group and the 22q11.2DS group correspond to a decreased activation in the posterior region. Importantly when considering left and right disparity between the AH and 22q11.2DS groups no evidence of interhemispheric specialization emerged. Whereas the anterior region responds to self-centered mental imagery strategies, the posterior region is involved in successful episodic memory retrieval (Cabeza and Nyberg, 2000; Cavanna and Trimble, 2006). Episodic memory relies on the ability to remember past experiences (Tulving, 1972) with autobiographical references (Tulving, 1983) and plays a role in mental imagery (Tulving, 1983; Cabeza and Nyberg, 2000; Rubin et al., 2003; Daselaar et al., 2008). Clinical data have pointed out to a link between impaired episodic memory and auditory verbal hallucinations (Seal et al., 2004; Badcock et al., 2005; Berenbaum et al., 2008; Daselaar et al., 2008).

From a structural point of view, a significant volume reduction of the parietal lobe has been described in 22q11.2DS (Schaer et al., 2010). Results from functional connectivity also show atypical connectivity involving the left precuneus and PCC regions during resting state (Debbane et al., 2012). Concerning patients with hallucinations however, no clear alterations of the precuneus have been identified as far as we know (Allen et al., 2008).

In addition to the BA 19 and posterior parietal similarities, the at-risk groups showed unique differences in comparison to the controls. Compared to the control group decreased activation in the left parieto-occipital junction and the right posterior temporal BA39 was found in the 22q11.2DS group, but not in the AH group. This finding might correspond to a diminished salience for other related cues in 22q11.2DS. As mentioned in the last section, posterior parietal cortex, especially the posterior part of the precuneus, is particularly involved in successful retrieval of episodic memory (Wagner et al., 2005; Cavanna and Trimble, 2006; Elman et al., 2013) and in remembering familiar people (Maddock et al., 2001). The right temporo-occipital region (BA39) has been implicated in face processing (Puce et al., 1995; Dichter et al., 2009) and in the increased attention to salient social information because of its interactive processing with emotional information (Norris et al., 2004). Interestingly, it has recently been shown that the right temporo-occipital region presents decreased activation in response to affective social versus affective non-social images in schizophrenia (Bjorkquist and Herbener, 2013). Clinical data indicates that the 22q11.2DS syndrome exposes to an increased risk of social withdrawal, poor social functioning and emotion recognition deficits (Baker and Skuse, 2005; Debbane et al., 2006; Campbell et al., 2009).

**Figure 3:**
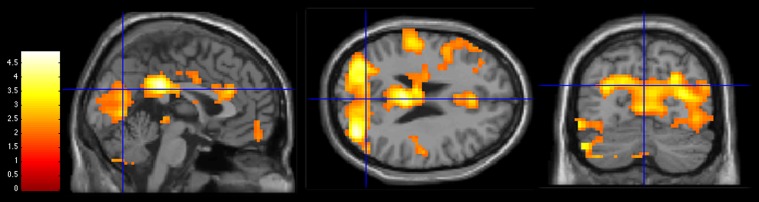
**Activations during 3PP > 1PP contrast in group comparisons (Control group > AH group) at a statistical threshold of *p* < 0.05**. Slice views at MNI coordinates (*x* = 0, *y* = −70, *z* = 25). The bar on the left shows the range of *T*-values.

**Figure 4:**
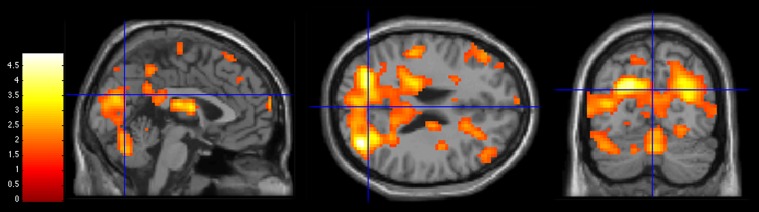
**Activations during 3PP > 1PP contrast in group comparisons (Control group > 22q11.2DS group) at MNI coordinates (*x* = 0, *y* = −70, *z* = 25)**. Statistical threshold of *p* < 0.05. The bar on the left shows the range of *T*-values.

In summary, our results show that both groups at risk (clinical and genetic) for hallucinations exhibited decreased activation in the parieto-occipital region during 3PP compared to the Control group (see Figures 3 and 4), which has been related to self-other distinction of imagined actions. We suggest that an impaired shift perspective and/or episodic memory dysfunctions might alter self-other distinction in hallucination-prone subjects. Consequently, the lack of reliable representations of the actions performed by others could account for SM action impairments previously observed by Larøi et al. (2005) and Debbane et al. (2008). Our results also argue in favor of a decreased salience toward others in the 22q11.2DS, as illustrated by the decreased activations in regions sustaining social cognition and episodic memory.

### At-risk groups: differences between the AH group and the 22q11.2DS group

We only obtained results for the prime condition when comparing the AH group and the 22q11.2DS group. This comparison indicated that the salience of self-other priming cues was different between the two groups at-risk for hallucinations, whereas no significant findings emerged for the 1PP and 3PP contrasts.

Compared to the AH group, adolescents with 22q11.2DS exhibited decreased activations of the caudate body, anterior cingulate BA32 and right superior frontal BA 10 for the prime self > prime other contrast. In line with our results, a significant lower level of activation was found in the caudate nucleus and the anterior cingulate cortex during self-reflective processing in adolescents with 22q11.2DS (Schneider et al., 2012). Several meta-analyses have highlighted the role of the anterior cortical midline structure and especially the anterior cingulate cortex in self-specific stimuli processing (Van Der Meer et al., 2010; Murray et al., 2012; Qin et al., 2012). It has also been shown that the caudate nucleus and the anterior cingulate cortex are engaged in reward and personal relevance, i.e., valuing external and internal stimuli with regard to their meaning for the subject (Enzi et al., 2009).

Our results might therefore reflect a decreased salience toward self-related cues in the 22q11.2DS compared to the AH group. The differences between the two groups could be related to neuro-structural alterations in the 22q11.2DS. Indeed reduced volume grey matter and cortical thickness have been described in the anterior cingulate cortex (Dufour et al., 2008; Bearden et al., 2009) and several studies have shown an increased volume of the caudate nucleus (Eliez et al., 2002; Kates et al., 2004; Gothelf et al., 2007). However according to several studies these regions are relatively spared in adolescents and adults with schizotypal traits (Spencer et al., 2007; Moorhead et al., 2009; Ettinger et al., 2012).

Compared to the AH group, adolescents with 22q11.2DS exhibited decreased activations in the right postcentral gyrus BA3 (somatosensory cortex S1) and anterior prefrontal cortex BA10 for prime other > prime self-contrast. The decreased activation of the somatosensory cortex in the 22q11.2DS group for the prime other > prime self contrast is in contradiction with previous work indicating that this region responds specifically to 1PP (Ruby and Decety, 2001, 2003, 2004). In our task however the role of the right somatosensory cortex and the anterior prefrontal cortex BA10 might be related respectively to emotion processing (Adolphs et al., 2000; Pourtois et al., 2004; Hooker et al., 2008; Saxbe et al., 2012) and mental states attribution (Gilbert et al., 2006; Burgess et al., 2007; Benoit et al., 2010). The reduced activations in these regions are in line with clinical evidence showing impairments in cognitive theory of mind tasks in 22q11.2DS (Chow et al., 2006; Campbell et al., 2011; Ho et al., 2012).

In summary, in comparison to subjects at clinical risk, adolescents with 22q11.2DS showed atypical patterns of activations when primed for themselves and their best friend. More precisely, decreased activations were found in regions involved in self-relevance, emotion processing and attribution.

## Limitations

The present study must be considered with limitations. First, the restricted sample sizes make it difficult to completely exclude the absence of significant results for the 1PP > 3PP contrasts. Future studies with increased statistical power could address this issue. Concerning the group selection, the 22q11.2DS group had lower IQ scores compared to the Control group. However, the behavioral results showed that response times and difficulty ratings did not significantly differ between groups. This suggests that 22q11.2DS subjects were not put in a more difficult position due to the intellectual deficits they might present.

The functional imaging paradigm did not include a cognitive control for the prime and action simulation period other than the perspective-taking variants, which could be included in a future version of this paradigm.

Future studies should address the neurodevelopmental issues of action simulation during adolescence by also comparing children and adults data or data with longitudinal follow-up. More research exploring shift perspective and agency processes may further contribute to a better understanding of action misattribution biases in hallucination-prone subjects.

## Conclusion

This study constitutes the preliminary step of a neuroscientific examination targeting the neural correlates of self-other discrimination in mental imagery for hallucination-prone adolescents. We suggest that impairment in the capacity to shift perspective and/or episodic memory dysfunction may alter self-other distinction in hallucination-prone subjects.

### Conflict of interest statement

The authors declare that the research was conducted in the absence of any commercial or financial relationships that could be construed as a potential conflict of interest.
