# Supplementary material for: Action simulation in hallucination-prone adolescents
Source: Front Hum Neurosci. 2013 Jul 4;7:329. doi: 10.3389/fnhum.2013.00329 (PMC3701149; doi:10.3389/fnhum.2013.00329)
Supplement: Supplementary file 1 [file DataSheet1.DOC]

Modèle linéaire général
Facteurs intra-sujets	
Mesure: MEASURE_1	
evaluation	Variable dépendante	
1	Eval_Other	
2	Eval_Self	

Facteurs inter-sujets	
	N	
groupe	1.00 = Control group	22	
	2.00 = AH group	12	
	3.00 = 22q group	13	

Descriptive statistics	
	groups	Mean	Standard deviation	N	
Eval_Other	Control group	1.7218	.38859	22	
	AH group	2.0608	.45332	12	
	22q group	1.8738	.57209	13	
	Total	1.8504	.47230	47	
Eval_Self	Control group	1.5882	.34366	22	
	AH group	1.7933	.34089	12	
	22q group	1.7381	.52847	13	
	Total	1.6820	.40360	47	

Tests multivariés	
Effet	Valeur	D	ddl de l'hypothèse	Erreur ddl	Sig.	
evaluation	Trace de Pillai	.232	13.315	1.000	44.000	.001	
	Lambda de Wilks	.768	13.315	1.000	44.000	.001	
	Trace de Hotelling	.303	13.315	1.000	44.000	.001	
	Plus grande racine de Roy	.303	13.315	1.000	44.000	.001	
evaluation * groupe	Trace de Pillai	.033	.752	2.000	44.000	.477	
	Lambda de Wilks	.967	.752	2.000	44.000	.477	
	Trace de Hotelling	.034	.752	2.000	44.000	.477	
	Plus grande racine de Roy	.034	.752	2.000	44.000	.477	

Test de sphéricité de Mauchly	
Mesure: MEASURE_1	
Effet intra-sujets	W de Mauchly	Khi-deux approché	ddl	Sig.	Epsilon	
					Greenhouse-Geisser	Huynh-Feldt	Borne inférieure	
evaluation	1.000	.000	0	.	1.000	1.000	1.000	


Tests des effets intra-sujets	
Mesure: MEASURE_1	
Source	Somme des carrés de type III	ddl	Moyenne des carrés	D	Sig.	
evaluation	Sphéricité supposée	.701	1	.701	13.315	.001	
	Greenhouse-Geisser	.701	1.000	.701	13.315	.001	
	Huynh-Feldt	.701	1.000	.701	13.315	.001	
	Borne inférieure	.701	1.000	.701	13.315	.001	
evaluation * groupe	Sphéricité supposée	.079	2	.040	.752	.477	
	Greenhouse-Geisser	.079	2.000	.040	.752	.477	
	Huynh-Feldt	.079	2.000	.040	.752	.477	
	Borne inférieure	.079	2.000	.040	.752	.477	
Erreur(evaluation)	Sphéricité supposée	2.315	44	.053			
	Greenhouse-Geisser	2.315	44.000	.053			
	Huynh-Feldt	2.315	44.000	.053			
	Borne inférieure	2.315	44.000	.053			


Tests des contrastes intra-sujets	
Mesure: MEASURE_1	
Source	evaluation	Somme des carrés de type III	ddl	Moyenne des carrés	D	Sig.	
evaluation	Linéaire	.701	1	.701	13.315	.001	
evaluation * groupe	Linéaire	.079	2	.040	.752	.477	
Erreur(evaluation)	Linéaire	2.315	44	.053			
